# Cancer-Related Fatigue in Head and Neck Cancer Survivors: Longitudinal Findings from the Head and Neck 5000 Prospective Clinical Cohort

**DOI:** 10.3390/cancers15194864

**Published:** 2023-10-05

**Authors:** Linda Sharp, Laura-Jayne Watson, Liya Lu, Sam Harding, Katrina Hurley, Steve J. Thomas, Joanne M. Patterson

**Affiliations:** 1 Population Health Sciences Institute, Newcastle University Centre for Cancer, Newcastle University, Newcastle NE1 7RU, UK; liya.lu@nhs.scot; 2 Speech & Language Therapy, Sunderland Royal Hospital, South Tyneside and Sunderland NHS Foundation Trust, Sunderland SR4 7TP, UK; laura-jayne.watson1@nhs.net; 3 Speech and Language Therapy Research Unit, Southmead Hospital North Bristol NHS Hospital Trust, Bristol BS10 5NB, UK; samantha.harding@nbt.nhs.uk; 4 Head & Neck 5000 Study, Weston NHS Foundation Trust, University of Bristol, Bristol BS8 1TU, UKsteve.thomas@bristol.ac.uk (S.J.T.); 5 Liverpool Head and Neck Centre, School of Health Science, University of Liverpool, Liverpool L69 3BG, UK; joanne.patterson@liverpool.ac.uk

**Keywords:** fatigue, head and neck cancer, oral cavity cancer, oropharynx cancer, larynx cancer, HN5000, psycho-oncology

## Abstract

**Simple Summary:**

Cancer-related fatigue (CRF) is a common side-effect of cancer and its treatments, but few studies have investigated CRF in head and neck cancer. Using data from 2847 patients included in the Head and Neck 5000 prospective clinical cohort, we investigated CRF over 12 months from cancer diagnosis. At baseline, shortly after diagnosis, 27.8% of patients had CRF. This rose to 44.7% at 4 months and fell to 29.6% at 12 months. In adjusted models, the likelihood of having CRF over 12 months was significantly higher in patients who were female, current smokers, and had comorbid conditions or depression at baseline. It was also higher in patients with stage 3 or 4 disease and who had multimodal treatment. The high prevalence of CRF indicates that there is a need for additional interventions and supports for affected HNC patients; these could be targeted towards patients in the higher risk groups.

**Abstract:**

Cancer-related fatigue (CRF) is a common side-effect of cancer and its treatments. For head and neck cancer (HNC), CRF may exacerbate the symptom burden and poor quality-of-life. Using data from the Head and Neck 5000 prospective clinical cohort, we investigated clinically important CRF over a year post-diagnosis, assessing temporal trends, CRF by HNC site and treatment received, and subgroups at higher risk of CRF. Recruitment was undertaken in 2011–2014. Socio-demographic and clinical data, and patient-reported CRF (EORTC QLQ-C30 fatigue subscale score ≥39 of a possible 100) were collected at baseline (pre-treatment) and 4- and 12- months post-baseline. Mixed-effects logistic multivariable regression was used to investigate time trends, compare cancer sites and treatment groups, and identify associations between clinical, socio-demographic and lifestyle variables and CRF. At baseline, 27.8% of 2847 patients scored in the range for clinically important CRF. This was 44.7% at 4 months and 29.6% at 12 months. In the multivariable model, after adjusting for time-point, the odds of having CRF over 12 months were significantly increased in females and current smokers; those with stage 3/4 disease, comorbidities and multimodal treatment; and those who had depression at baseline. The high prevalence of clinically important CRF indicates the need for additional interventions and supports for affected HNC patients. These findings also identified patient subgroups towards whom such interventions could be targeted.

## 1. Introduction

Head and neck cancer (HNC) is an umbrella term for tumours that arise in the oral cavity, pharynx, larynx, nasal cavity and sinuses. The complexity of the disease means that patients often undergo aggressive multi-modal treatment [1]. This treatment, and the location of the tumours, can mean that many functions of everyday life, such as speech, swallowing and breathing, are adversely impacted [2,3]. Consequently, post-treatment these patients often have high levels of psychological distress which, in turn, results in poor quality-of-life (QoL) [4,5].

Cancer-related fatigue (CRF) is one of the most common side-effects of cancer, affecting up to 90% of patients at some point [6]. It has been defined as a subjective sense of physical, emotional and/or cognitive tiredness or exhaustion related to cancer or its treatment that is distressing, persistent, and not proportional to recent activity [7]. It is generally not relieved by sleep or rest and can have a detrimental impact on emotional, physical and cognitive functioning, social activities and QoL [8]. For HNC patients, who may already have a significant symptom burden, this impact may be especially pronounced. Two qualitative studies in HNC suggest that CRF can act as a barrier to everyday functioning including eating and drinking, especially for those who have dysphagia and for whom swallowing feels effortful [9,10]. In terms of prevalence, it has been reported that 85% of HNC patients with locally advanced disease had CRF [11]. Another study of patients who had chemoradiation found CRF peaked 1–2 weeks post-radiation and remained higher than baseline for up to two years in half of the patients [12]. Predictors of CRF in HNC include radiotherapy dose and volume, pre-treatment depression, time since treatment and younger age [13,14,15]. However, available studies are limited in number. They mostly used a cross-sectional design, were from single centres and had very modest sample sizes, raising concerns about the robustness and generalisibility of the results.

One of the research challenges in CRF is that some instruments used to measure it generate a “score”, but how those scores should be interpreted for individual patients or groups of patients at a single point in time is unclear. The European Organization for Research and Treatment of Cancer Quality of Life Questionnaire C30 (EORTC QLQ-C30)—a well-validated measure of functioning and QoL in cancer—includes a subscale which can be used to generate a fatigue score [16]. In 2016, Giesinger and colleagues [17] established thresholds for clinically important fatigue for the EORTC QLQ-C30. This means that it is now possible to use this instrument to identify patients with clinically important CRF who may require further exploration and/or health professional intervention.

Using data from the Head and Neck 5000 (HN5000) prospective, longitudinal, clinical cohort study [18,19], we aimed to investigate clinically important CRF over the first year after diagnosis with HNC. Our specific objectives were to (1) assess temporal trends, (2) compare CRF across HNC sites and by treatment received, and (3) identify subgroups at higher risk of CRF.

## 2. Materials and Methods

### 2.1. Study Population

The protocol and HN5000 population have been reported elsewhere [18,19]. In brief, people aged ≥16 years with a new diagnosis of HNC at 76 National Health Service (NHS) hospitals in England, Scotland and Wales were invited to participate. Those who lacked capacity to provide informed consent or who were deemed by their clinical team to be too vulnerable for participation were ineligible. Participants provided written informed consent and the study was approved by the National Research Ethics Committee (South West Frenchay Ethics Committee, reference number 10/H0107/57, 5 November 2010) and the Research and Development departments of participating NHS Trusts.

The dataset made available for analysis (version 2.1) included 5404 individuals who consented to participate over April 2011–December 2014. For this analysis, we defined the study population as comprising people who had been diagnosed with oral cavity, oropharynx, larynx, hypopharynx, thyroid or salivary gland cancer, identified using ICD10 codes. We further limited consideration to individuals who had received treatment (curative or non-curative) and did not have recurrent disease during follow-up (*n* = 3779) (Supplementary Figure S1). Due to small numbers, patients with cancers of the hypopharynx were combined with those with laryngeal cancers for analysis.

### 2.2. Procedures

Participants completed a health and lifestyle survey at baseline (before treatment started) and a series of standard questionnaires at 4- and 12-months post-baseline. The baseline survey collected data on socio-demographic variables (e.g., marital status) and lifestyle (e.g., smoking status, alcohol consumption in past week). The questionnaires included the EORTC QLQ-C30 [16] and the Hospital Anxiety and Depression Scale (HADS), a validated screening tool for anxiety and depression [20].

Information abstracted from participants’ medical records at baseline included patient characteristics (e.g., age, sex, ethnicity); ICD10 tumour site [21]; TNM stage (seventh edition) [22]; laterality of primary tumour; and comorbidities (classified using the Adult Comorbidity Evaluation (ACE-27)) [23]. Serological human papilloma virus (HPV) status at baseline was defined as positive where HPV16E6 was >1000 median fluorescence intensity [24]. Treatments received by 4 months were abstracted from medical records. A deprivation category based on the English Index of Multiple Deprivation 2010 [25] was assigned based on each participant’s home address at baseline.

### 2.3. Outcome Variable

For this study, we focussed on the fatigue subscale (“FA”) of the EORTC QLQ-C30, which includes three questions on fatigue experienced over the past week. Question responses were combined and linearly transformed into a score in the range 0–100, treating missing data as recommended [26]; higher scores represent more severe CRF. A FA subscale score of ≥39 out of a possible 100 indicates the presence of clinically important CRF [17]. From the subscale scores, we created a binary outcome variable representing, for each patient, the presence or absence of clinically important CRF. This was performed for each time-point, namely baseline, 4 and 12 months.

### 2.4. Statistical Analysis

To be included in the analysis of CRF, individuals in the study population had to have completed the FA subscale questions at baseline (Supplementary Figure S1). To assess potential bias, the characteristics of those who completed the FA subscale (*n* = 2847) and those who were in the study population but did not complete the subscale (*n* = 932), were compared using chi-square tests.

The proportions of participants with clinically important CRF at each time-point—baseline, 4 and 12 months—were computed overall, by cancer site and by treatment received; the analysis of treatment was limited to the 4- and 12-month time-points. Mixed-effects multivariable logistic regression was used to examine time trends and identify factors associated with presence of clinically important CRF over time (i.e., across all three time-points). These models are particularly appropriate for longitudinal data as they allow for inclusion of all surveys completed by each individual, taking within-subject correlations into account and producing robust error estimates [27]. First, we assessed bivariate associations between each potential predictor variable and the outcome (adjusting for time-point). The potential predictor variables, shown in Table 1, comprised the socio-demographic characteristics, lifestyle factors, clinical variables and presence of significant depression at baseline. Significant depression was defined as score of ≥8 on the HADS depression subscale, following recommendations for detecting depression in cancer survivors [28]. Time-points and variables significant at the 5% level were included in an initial multivariable model. Wald tests were then used to reduce the model. The final model included variables that remained significant at the 5% level when adjusted for other variables. In terms of interpretation, the model provides estimates of the effect of each variable over all time-points (i.e., over the 12-month follow-up period). We used Akaike’s Information Criterion (AIC) to compare models with random intercept and random slope with those including random intercept only. There was little difference in AIC so we fitted the least complex option: random intercept with structured covariance. We excluded people with missing data if <3% of individuals had missing data for the variable; if ≥3% were missing, we included an “unknown” category of the variable. STATA version 16 was used for analysis and *p* ≤ 0.05 (two-sided) was considered statistically significant throughout.

## 3. Results

### 3.1. Participants

There were no differences in age-group, sex, ethnicity and stage between the 2487 participants included in the analysis and the 932 HN5000 recruits in the study population who did not complete the CRF questions at baseline. There were statistically significant differences in deprivation, cancer site and comorbidities. The analysis dataset included fewer deprived patients with fewer comorbidities and more patients with thyroid and salivary gland cancers..

Of the 2847 patients in the analysis dataset, 704 (24.7%) had cancer of the oral cavity, 1119 (39.3%) oropharyngeal cancer, 786 (24.8%) laryngeal or hypopharynx cancer, 186 (6.5%) thyroid cancer and 132 (4.6%) cancer of the salivary glands (Table 1). More than 70% were men; almost half were aged 50–64 at recruitment and 37% were 65 or older. Just over one-quarter had stage 1 disease, 17% stage II, 14% stage III and 41% stage IV. Almost 10% had a bilateral tumour. One-fifth had moderate/severe decompensation on the comorbidity index.

### 3.2. CRF at Each Time-Point

At baseline, 27.8% of participants scored in the range for clinically important CRF. This rose to 44.7% at 4 months and declined (although not to baseline levels) to 29.6% at 12 months (Table 2). When examined by site, at baseline the prevalence was highest (32.2%) in those with thyroid tumours, followed by those with oral cavity and larynx tumours (29.5%); the lowest prevalence was in those with oropharynx (25.2%) and salivary gland tumours (25.0%). All cancer groups had higher prevalence at 4 months, and this was particularly pronounced for oropharynx tumours, with more than half (54.4%) of patients having clinically important CRF. At 12 months, just over 30% of those with oropharynx, larynx and salivary gland tumours had CRF.

When considered by treatment received, at 4 months more than half of those who had multi-modal treatment had CRF (surgery + chemoradiotherapy, 57.7%; chemoradiotherapy, 56.1%; surgery and radiotherapy, 50.9%). The prevalence was much lower among those who had a single treatment modality (surgery, 31.4%; radiotherapy 37.5%). The prevalence of CRF declined at 12 months, but was still 30% or higher in all treatment groups with the exception of those who had surgery alone (25.0%).

The prevalence of clinically important CRF by socio-demographic, lifestyle and other clinical factors at each time-point is shown in Supplementary Table S1.

### 3.3. Factors Associated with Clinically Important CRF over Time

In the multivariable mixed logistic regression model, the odds of clinically important CRF were four times higher at the 4-month time-point (OR = 4.05, 95% CI 3.24–5.08) than at baseline; at 12 months the OR was 1.67 (95% CI 1.37–2.03) (Table 3). Depression at baseline was associated with a greater than 14-fold increase in clinically important CRF over 12 months after adjusting for other variables (OR = 14.7, 95% CI 8.81–24.6). The odds of clinically important CRF were twice as high in female than male patients, and while there was no trend in odds for deprivation, those resident in the most-deprived areas had 59% higher odds (95% CI 1.05–2.41) of clinically important CRF than those resident in the least-deprived areas. Two lifestyle factors were significantly associated with clinically important CRF over time: current smokers had an OR of 1.79 (95% CI 1.19–2.72) compared to never smokers; and the odds decreased with an increasing number of days per week the individual drank alcohol (drinking alcohol 3–7 days/week vs none, OR = 0.57, 95% CI 0.42–0.78). In terms of clinical variables, the odds of clinically important CRF increased with increasing comorbidities; were significantly higher in those with stage 3 or stage 4 disease than those with stage 1 disease; and were highest in those who had surgery and radiotherapy (surgery and radiotherapy vs. surgery alone: OR = 1.71, 95% CI 1.13–2.58).

## 4. Discussion

This is the first large-scale longitudinal study to investigate CRF in HNC patients, and the predictors of this over time. We found that a more advanced tumour stage, multimodal treatment, female sex and being a smoker were all associated with a higher odds of CRF over 12 months. Additionally, those who had depression and more co-morbidities at baseline (before treatment commenced) were more likely to have CRF over 12 months.

One of the most striking findings was the high prevalence of CRF in this cohort. Forty-five percent of patients overall, and more than half of those with oropharyngeal cancer, scored in the range for CRF at 4-month follow-up, while the prevalence fell afterwards, with 30% having CRF at 12-month follow-up. The higher prevalence at 4 months could perhaps be explained by the fact that some patients would have still been undergoing systemic treatments, during which patients often experience severe fatigue [29]. This is supported by the observation that the group with the lowest prevalence at 4 months was those who had surgery alone (31.4%). In terms of the particularly high prevalence at 4 months in those with oropharyngeal tumours, this may be because patients with these tumours tend to be younger and fitter at diagnosis than patients with, for example, laryngeal tumours; thus, they may experience a greater, or more obvious, decline in function at 4 months. In addition, more than half of patients with oropharyngeal cancers are HPV positive [30], and past research (albeit a study limited to 94 patients) found that patients with HPV-related squamous cell HNC more often had increases in fatigue during treatment than those with tumours unrelated to HPV [31]. In that study, during the treatment similar increases were also seen in inflammatory markers (such as interleukin-6 (IL-6)) in the group with HPV-related tumours. This is consistent with research on mechanisms of CRF, which suggests an important role for proinflammatory cytokines, and the pathways to which these contribute [8]. Why these pathways might be more active in oropharyngeal, or HPV-related, tumours requires further investigation.

Several predictors of CRF have been identified in the current study. The strongest relationship—a 14-fold increase in odds—was found among those who had depression at baseline. Depression was assessed using self-completion of the HADS, which has high sensitivity and specificity compared to semi-structured interviews with patients [32]. The prevalence of CRF at each time-point was very high among the patients with depression at baseline: 68%, 79% and 64% at baseline, 4 months and 12 months, respectively. However, it is worth noting that a substantial proportion of patients without depression at baseline also had clinically important CRF at each time-point, namely, 19%, 39% and 23%. This highlights the importance of not assuming that CRF is simply a function or consequence of a pre-existing depression in HNC patients or, indeed, is likely to occur only in patients with a history of depression.

A previous study of 70 HNC patients reported a correlation between CRF and depression before, during and shortly after chemoradiation [13]; another cross-sectional study of 58 patients reported a similar link [15]. Our analysis extends these findings by including a much larger population, adjusting for confounders, and showing that the association persists over (at least) 12 months post-diagnosis. Research among survivors with other cancers also reports associations between depression and CRF [33]. Psychosocial stressors, such as depression, promote inflammation [34] and this suggests a potential explanation for the observed relationship. Research seeking to better understand the (likely complex) inter-relationships between depression and CRF over time among cancer survivors, and underlying mechanisms, would be of value.

The higher odds of CRF among female patients observed here is consistent with findings from a meta-analysis of 24 studies of CRF measured at various time-points in a range of cancer sites [33]; that analysis reported a two-fold increased risk in females, similar to the magnitude of effect seen here. The authors of the meta-analysis postulated that the association could be due to smoking which (as we observed) has been associated with CRF and is more prevalent in men in many populations. However, our finding for sex was adjusted for smoking, suggesting that is unlikely to be the explanation. In terms of alternative explanations, one possibility could be higher underlying higher levels of inflammation among women. In a study that involved the clustering of cancer symptoms and cytokine levels, female patients were over-represented in groups with moderate/high fatigue; those groups also had significantly higher levels of proinflammatory cytokine IL-6 [35]. The sample size of that study was modest, and the findings require confirmation. However, it is also worth noting that, while fatigue in people without cancer is not the same as CRF, in the general population fatigue is more common in women [36].

In terms of clinical variables, a previous study of 140 HNC patients on average 3–4 months after the completion of treatment, reported that fatigue was more likely to be identified as a concern by those with advanced disease and who had had radiotherapy and/or chemotherapy [37]. This has some parallels with our analysis, which found that stage III or IV disease (versus stage I) and radiotherapy or multi-modal treatment (versus surgery only) were associated with increased odds of CRF over time. Importantly, the association reported here between treatment and CRF was adjusted for disease stage (and vice versa). Studies in other cancers suggest a high prevalence of CRF persisting into long-term survivorship (see, for example, [38,39,40]). Longer-term follow-up data for the HN5000 cohort is accruing and would be valuable to track the ongoing trajectory of CRF as time passes from the completion of treatment.

One question raised by our findings is the extent to which the fatigue is a result of the cancer, the treatment or some other factor (and would therefore have been present in the absence of cancer). The lack of pre-diagnosis data and a non-cancer comparator population means that it is impossible to know to what extent cancer and/or its treatment presents an “excess risk” of fatigue. Some studies in other cancers have shown slightly higher mean levels of CRF (as measured using the EORTC QLQ-C30) in survivors than population controls [41,42], but whether this also holds in HNC is unclear. In addition, while our analysis took account of all questionnaires completed by each participant over time, the approach did not distinguish between different subgroups of survivors, who might have had different patterns of CRF over time (e.g., those with persistently high CRF vs. those where CRF peaked during/following treatment then declined). In other cancers, researchers have shown that different subgroups of patients may have different trajectories of QoL, wellbeing and fear of recurrence over time [43,44]. Further research using, for example, group-based trajectory methods [45], would be worthwhile to determine whether there are distinct temporal trajectories of CRF in HNC and whether these, in turn, have different predictors.

### 4.1. Implications

Across cancer as a whole, there is a growing evidence-base around interventions for CRF [46,47,48] and this is beginning to be translated into guidelines. The NCCN 2020 guidelines recommend a graded intervention, starting with education and counselling, then physical activity, psychosocial interventions and, if needed, pharmacological interventions [7]. However, a recent editorial observed that limited progress has been made in the clinical assessment and treatment of CRF in HNC, suggesting that this may be because this symptom is “lost” in the management of the other complex medical problems these patients may have [49]. Indeed our own studies in the same patient cohort highlighted HNC-specific problems and functional impairments, including with swallowing, social contact and social eating [50,51]. Qualitative work among HNC patients indicates that they perceive a lack of support for CRF post-treatment [52]; this was confirmed in a survey of HNC survivors among whom fatigue was one of the most frequent unmet needs [53]. The results reported here further emphasize how important it is that clinical teams put greater emphasis on this debilitating problem and screen HNC patients for CRF, offering, where appropriate, support and intervention. It is important to be cognisant of the probability of functional impairments occurring alongside CRF, which implies that patients will likely need coordinated support and complex interventions from a range of healthcare professionals to help them manage a constellation of longer-term treatment side-effects. In relation to screening for CRF specifically, a standardised tool could be used either in the pre- or post-treatment setting, and there are many available [54]. It may also be of value to seek to distinguish between those patients for whom significant fatigue arose for the first time following the cancer diagnosis and/or treatment and those who experienced significant fatigue pre-cancer; it is possible that these groups may benefit from different interventions or mitigation strategies. An alternative approach could be to target the offer of support towards patients in the groups shown here to be at higher risk of CRF (e.g., women, those with depression).

In terms of specific interventions for CRF in HNC, ongoing research is starting to explore the feasibility and acceptability of exercise and/or physical activity interventions (https://www.wcrf.org/researchwefund/fitness-patients-chemo-radiotherapy-head-neck-cancer/, accessed on 17 June 2023; https://www.isrctn.com/editorial/retrieveFile/d9ef52ed-595e-4d35-aca6-446b4bedd973/42265, accessed on 17 June 2023). Should the findings from such studies be positive, it is possible that routine implementation of physical activity interventions before, during or after treatment could have benefits in terms of CRF for some patients.

Our results highlight the importance of identifying, at the pre-treatment stage, patients’ smoking status to improve timely access to smoking cessation services. It could be helpful to explain to patients who smoke that, if they were willing to engage with behaviour change, this could potentially reduce their chances of CRF. Similarly, the observed association between comorbidities and CRF indicates that HNC management should include ensuring that patients’ other conditions are optimised.

### 4.2. Strengths and Limitations

In terms of strengths, the HN5000 cohort provided a large sample, recruited from multiple UK centres. This enabled a comprehensive analysis of the trajectory of CRF in the first year following diagnosis; most past studies have been limited to single centres, with relatively small sample sizes and/or cross-sectional designs. The EORTC QLQ-C30 is a widely used and well-validated cancer-specific health-related QoL questionnaire, but the FA subscale includes only three questions and does not distinguish between emotional, physical and cognitive CRF. In addition, HN5000 participants were disproportionality at an earlier cancer stage, compared to the entire HNC population. Thus, the observed association between more advanced stage cancer and CRF, and the differences between those included in the analysis and those who did not complete the CRF questions at baseline means we have likely under-estimated the true prevalence of CRF in HNC patients. However, there is no reason to believe internal comparisons within the cohort are biased. Finally, the lack of an external age- and sex-matched non-cancer comparator population is a limitation.

## 5. Conclusions

Almost one-third of HNC patients report clinically important CRF at 12-months post-diagnosis. The odds of CRF over 12-months are increased in those with significant depression pre-treatment and in women, smokers and those with more advanced disease who have multi-model treatment and other comorbidities. These findings indicate the need for additional focus during HNC follow-up on this debilitating symptom and for the implementation of interventions to alleviate CRF.

## Figures and Tables

**Table 1:** Baseline characteristics of study population: non-recurrent oral cavity, oropharynx, larynx, hypopharynx, thyroid, salivary glands cancer patients who had received treatment ^1^ (*n* = 2847).

Characteristic	*n* (%)	Characteristic	*n* (%)
*Age at date of consent (years)*		*Significant depression* ^2^	
<50	443 (15.6)	No	2325 (81.7)
50–64	1341 (47.1)	Yes	507 (17.8)
≥65	1063 (37.3)	Unknown	15 (0.5)
*Sex*		*Tumour site*	
Male	2027 (71.2)	Oral cavity	704 (24.7)
Female	820 (28.8)	Oropharynx	1119 (39.3)
*Ethnicity*		Larynx/hypopharynx	706 (24.8)
White	2677 (94.0)	Thyroid	186 (6.5)
Other	86 (3.0)	Salivary glands	132 (4.7)
Unknown	84 (3.0)	*Stage*	
*Marital status*		I	788 (27.7)
Married/cohabiting	1910 (67.1)	II	482 (16.9)
Other	837 (29.4)	III	389 (13.7)
Unknown	100 (3.5)	IV	1177 (41.3)
*Education*		Unknown	11 (0.4)
Primary	35 (1.2)	*Comorbidity index*	
Secondary	1145 (40.2)	No comorbidity	1304 (45.8)
Tertiary	1428 (50.2)	Mild decompensation	942 (33.1)
Unknown	239 (8.4)	≥Moderate decompensation	545 (19.1)
*Deprivation quintile*		Unknown	56 (2.0)
1 (least deprived)	520 (18.2)	*HPV16 E6*	
2	509 (17.9)	Negative	1725 (60.6)
3	608 (21.4)	Positive	760 (26.7)
4	455 (16.0)	Unknown	362 (12.7)
5 (most deprived)	502 (17.6)	*Side of primary tumour*	
Unknown	253 (8.9)	Unilateral	2564 (90.1)
*Smoking status*		Bilateral ^3^	274 (9.6)
Never	671 (23.6)	Unknown	9 (0.3)
Ex	1482 (52.1)	*Treatment received* ^4^	
Current	477 (16.7)	Surgery only	836 (29.4)
Unknown	217 (7.6)	Chemoradiotherapy only	781 (27.4)
*Alcohol consumed (days/week)*		Radiotherapy only	536 (18.8)
0	747 (26.3)	Surgery + radiotherapy	418 (14.7)
1–2	650 (22.8)	Surgery + chemoradiotherapy	276 (9.7)
3–7	1241 (43.6)		
Unknown	209 (7.3)		

^1^ And who completed fatigue subscale of QLQ-C30 at baseline; ^2^ score of ≥8 on HADS depression subscale; ^3^ includes midline, bilateral, left/midline and right/midline; ^4^ by 4 months; surgery only group includes 1 patient who had surgery and chemotherapy; chemoradiotherapy only group includes 5 patients who had chemotherapy only.

**Table 2:** Prevalence of clinically important CRF ^1^ at baseline, 4 and 12 months, overall and by tumour site and treatment received ^2^: number who completed subscale (N), number who scored in range for clinically important CRF (*n*) and percentages (%).

	Baseline			4 months			12 months		
	N	*n*	%	N	*n*	%	N	*n*	%
Overall	2847	791	27.8	2156	964	44.7	1957	580	29.6
*By site*									
Oral cavity	704	208	29.5	556	228	41.0	487	135	27.7
Oropharynx	1119	282	25.2	845	460	54.4	795	246	30.9
Larynx (and hypopharynx)	706	208	29.5	511	191	37.4	463	146	31.5
Thyroid	186	60	32.3	145	49	33.8	121	25	20.7
Salivary gland	132	33	25.0	99	36	36.4	91	28	30.8
*By treatment received*									
Surgery only	836	239	28.6	653	205	31.4	575	144	25.0
Chemoradiotherapy only	781	195	25.0	594	333	56.1	551	175	31.8
Radiotherapy only	536	159	29.7	381	143	37.5	353	113	32.0
Surgery + radiotherapy	418	124	29.7	320	163	50.9	296	94	31.8
Surgery + chemoradiotherapy	276	74	26.8	208	120	57.7	182	54	29.7

^1^ Score of ≥39 of a possible 100 on the FA subscale of the EORTC QLQ-C30; ^2^ at 4 months; surgery only group includes 1 patient who had surgery and chemotherapy; chemoradiotherapy only group includes 5 patients who had chemotherapy only.

**Table 3:** Multivariable logistic mixed regression results—significant predictors ^1^ of clinically important CRF over time ^2^: multivariable odds ratios (OR), 95% confidence intervals (95%CI), *p* values and Wald test *p* values.

Variable	OR (95% CI)	*p* Value	Wald Test *p*
*Time-point*			<0.001
Baseline	1		
4 months	4.06 (3.24–5.08)	<0.001	
12 months	1.67 (1.37–2.03)	<0.001	
*Sex*			<0.001
Male	1		
Female	2.33 (1.74–3.11)	<0.001	
*Deprivation quintile*			<0.001
1 (least deprived)	1		
2	1.09 (0.75–1.58)	0.647	
3	1.19 (0.83–1.71)	0.339	
4	0.79 (0.53–1.18)	0.254	
5 (most deprived)	1.59 (1.05–2.41)	0.028	
Unknown	1.55 (0.95–2.52)	0.079	
*Smoking status*			<0.001
Never	1		
Ex	1.12 (0.84–1.49)	0.449	
Current	1.80 (1.19–2.72)	0.006	
Unknown	1.18 (0.69–2.03)	0.549	
*Alcohol consumed (days/week)*			<0.001
None	1		
1–2	0.77 (0.54–1.09)	0.135	
3–7	0.57 (0.42–0.78)	<0.001	
Unknown	0.82 (0.46–1.46)	0.505	
*Significant depression*			<0.001
No	1		
Yes	14.72 (8.81–24.61)	<0.001	
*Stage*			0.004
I	1		
II	0.94 (0.65–1.37)	0.760	
III	1.62 (1.05–2.51)	0.029	
IV	1.48 (1.01–2.17)	0.044	
*Comorbidity index*			<0.001
No comorbidity	1		
Mild decompensation	1.81 (1.38–2.37)	<0.001	
≥Moderate decompensation	3.23 (2.24–4.67)	<0.001	
*Treatment received* ^3^			0.023
Surgery only	1		
Chemoradiotherapy only	1.49 (1.00–2.21)	0.049	
Radiotherapy only	1.40 (0.97–2.00)	0.069	
Surgery + radiotherapy	1.71 (1.13–2.58)	0.011	
Surgery + chemoradiotherapy	1.32 (0.80–2.16)	0.279	

^1^ Variables measured at baseline unless otherwise indicated; ^2^ a higher score indicates worse symptoms; ^3^ by 4 months; surgery only group includes 1 patient who had surgery and chemotherapy; chemoradiotherapy only group includes 5 patients who had chemotherapy only.

## Data Availability

The data that support the findings of this study are available from Head and Neck 5000. Further information may be found on the Head and Neck 5000 website: https://www.headandneck5000.org.uk/information-for-researchers/(accessed on 17 June 2023).

## Supplementary Materials

The following supporting information can be downloaded at: https://www.mdpi.com/article/10.3390/cancers15194864/s1, Figure S1: Flowchart showing study and analysis populations; Table S1: Prevalence of clinically important CRF1 at baseline, 4 and 12 months by socio-demographic, lifestyle and other clinical variables. Number who completed subscale (N), number who scored in range for clinically important CRF (*n*) and percentages (%).

## Abbreviations

CI	confidence interval
CRF	cancer-related fatigue
EORTC	European Organization for Research and Treatment of Cancer
FA	fatigue subscale of the EORTC QLQ-C30 questionnaire
HADS	Hospital Anxiety & Depression Scale
HN5000	Head and Neck 5000 study
HNC	head and neck cancer
HPV	human papilloma virus
IL	interleukin
NCCN	National Comprehensive Cancer Network
OR	odds ratio
QLQ	Quality of Life Questionnaire
QoL	quality of life
