# Supplementary material for: Cancer-Related Fatigue in Head and Neck Cancer Survivors: Longitudinal Findings from the Head and Neck 5000 Prospective Clinical Cohort
Source: Cancers (Basel). 2023 Oct 5;15(19):4864. doi: 10.3390/cancers15194864 (PMC10571913; doi:10.3390/cancers15194864)
Supplement: Supplementary file 1 [file cancers-15-04864-s001.zip › cancers-2484670-supplementary.pdf]

Cancer-Related Fatigue in Head and Neck Cancer Survivors: Longitudinal Findings from the Head and Neck 5000 Prospective Clinical Cohort

Linda Sharp, Laura-Jayne Watson, Liya Lu, Sam Harding, Katrina Hurley, Steve J. Thomas and Joanne M. Patterson

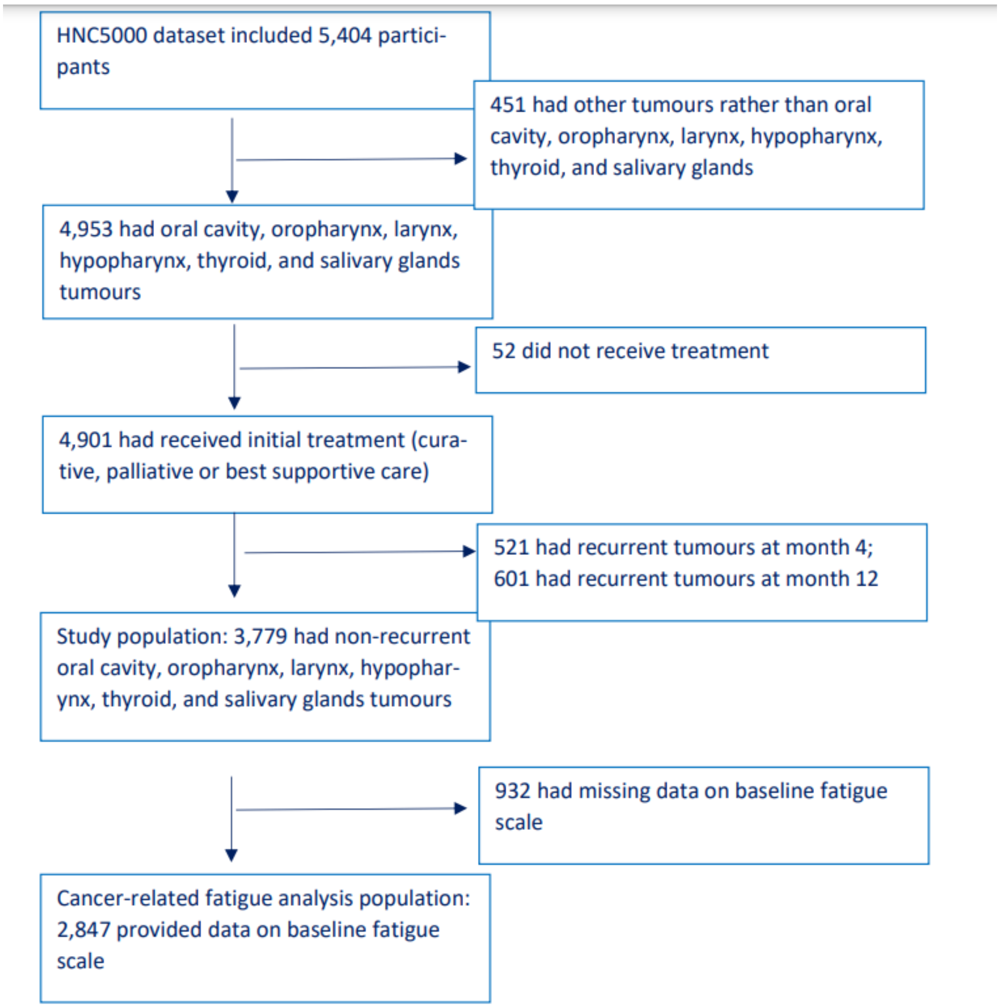

Figure S1. Flowchart showing study and analysis populations.

Table S1. Prevalence of clinically-important CRF[1] at baseline, 4 and 12 months, by socio-demographic, lifestyle, and other clinical variables at baseline: number who completed subscale (N), number who scored in range for clinically-important CRF (n) and percentages (%).

	Baseline			4 months			12 months		
	N	n	%	N	n	%	N	n	%
**Age at date of consent (years)**									
<50	443	151	34.1	311	152	48.9	257	76	29.6
50–64	1341	375	28.0	1012	490	48.4	943	305	32.3
≥65	1063	265	24.9	833	322	38.7	757	199	26.3
**Sex**									
Male	2027	506	25.0	1536	685	44.6	1390	390	28.1
Female	820	285	34.8	620	279	45.0	567	190	33.5
**Ethnicity**									
White	2677	738	27.6	2031	912	44.9	1853	544	29.4

Other	86	33	38.4	53	29	54.7	45	18	40.0
Unknown	84	20	23.8	72	23	31.9	59	18	30.5
**Marital status**									
Married/cohabiting	1910	493	25.8	1490	652	43.8	1363	368	27.0
Other	837	264	31.5	605	284	46.9	541	194	35.9
Unknown	100	34	34	61	28	45.9	53	18	34.0
**Education**									
Primary	35	13	37.1	24	11	45.8	22	11	50.0
Secondary	1145	361	31.5	850	393	49.2	758	251	33.1
Tertiary	1428	328	23.0	1126	475	42.2	1039	267	25.7
Unknown	239	89	37.2	156	85	54.5	138	51	37.0
**Deprivation quintile**									
1 (least deprived)	520	106	20.4	423	160	37.8	389	72	18.5
2	509	114	22.4	404	163	40.4	386	91	23.6
3	608	163	26.8	472	205	43.4	425	128	30.1
4	455	139	30.6	320	152	47.5	287	105	36.6
5 (most deprived)	502	194	38.7	347	192	55.3	303	131	43.2
Unknown	253	75	29.6	190	92	48.4	167	53	31.7
**Smoking status**									
Never	671	156	23.3	535	211	39.4	498	101	20.3
Ex	1482	397	26.8	1146	497	43.4	1054	310	29.4
Current	477	169	35.4	324	178	54.9	266	117	44.0
Unknown	217	69	31.8	151	78	51.7	139	52	37.4
**Alcohol consumed (days/week)**									
None	747	255	34.1	551	267	48.5	524	192	36.6
1-2	650	156	24.0	519	205	39.5	455	105	23.1
3-7	1241	305	24.6	955	419	43.9	859	234	27.2
Unknown	209	75	35.9	131	73	55.7	119	49	41.2
**Significant depression**[2]									
No	2325	442	19.0	1814	708	39.0	1643	381	23.2
Yes	507	345	68.1	334	254	76.1	307	196	63.8
Unknown	15	4	26.7	8	2	25.0	7	3	42.9
**Stage**									
I	788	222	28.2	601	189	31.5	534	132	24.7
II	482	130	27.0	370	153	41.4	329	106	32.2
III	389	107	27.5	309	133	43.0	279	88	31.5
IV	1177	330	28.0	870	486	55.9	808	251	31.1
Unknown	11	2	18.2	6	3	50.0	7	3	42.9
**Comorbidity index**									
No comorbidity	1304	264	20.3	1016	396	39.0	910	199	21.9
Mild decompensation	942	276	29.3	720	348	48.3	675	212	31.4
Moderate/severe decompensation	545	236	43.3	381	204	53.5	338	159	47.0
Unknown	56	15	26.8	39	16	41.0	34	10	29.4
**HPV16 E6**									
Negative	1725	497	28.8	1311	511	39.0	1182	358	30.3
Positive	760	174	22.9	579	316	54.6	554	151	27.3
Unknown	362	120	33.1	266	137	51.5	221	71	21.1
**Side of primary tumour**									
Unilateral	2564	701	27.3	1945	864	44.4	1771	514	29.0
Bilateral[3]	274	87	31.8	205	97	47.3	181	63	34.8
Unknown	9	3	33.3	6	3	50.0	5	3	60.0

[1] after linear transformation, possible scores may range from 0 to 100. A higher score indicates worse symptoms; [2] score of ≥8 on HADS depression subscale; [3] includes midline, bilateral, left/midline and right/midline.
